# Ventricular repolarization heterogeneity in patients with COVID‐19: Original data, systematic review, and meta‐analysis

**DOI:** 10.1002/clc.23767

**Published:** 2022-01-10

**Authors:** Elham Mahmoudi, Reza Mollazadeh, Pejman Mansouri, Mohammad Keykhaei, Shayan Mirshafiee, Behnam Hedayat, Mojtaba Salarifar, Matthew F. Yuyun, Hirad Yarmohammadi

**Affiliations:** ^1^ Gerash Amir‐al‐Momenin Medical and Educational Center Gerash University of Medical Sciences Gerash Iran; ^2^ Department of Cardiology, School of Medicine, Imam Khomeini Hospital Complex Tehran University of Medical Sciences Tehran Iran; ^3^ Tehran Heart Center Tehran University of Medical Sciences Tehran Iran; ^4^ Non‐Communicable Diseases Research Center (NCDRC), Endocrinology and Metabolism Research Institute Tehran University of Medical Sciences Tehran Iran; ^5^ VA Boston Healthcare System & Harvard Medical School Boston Massachusetts USA; ^6^ Division of Cardiology Columbia University New York New York USA

**Keywords:** COVID‐19, electrocardiography, QT dispersion, repolarization disparity, repolarization heterogeneity, sudden cardiac death, T‐peak to T‐end

## Abstract

**Background:**

Coronavirus disease‐2019 (COVID‐19) has been associated with an increased risk of acute cardiac events. However, the effect of COVID‐19 on repolarization heterogeneity is not yet established. In this study, we evaluated electrocardiogram (ECG) markers of repolarization heterogeneity in patients hospitalized with COVID‐19. In addition, we performed a systematic review and meta‐analysis of the published studies.

**Methods:**

QT dispersion (QTd), the interval between T wave peak to T wave end (TpTe), TpTe/QT (with and without correction), QRS width, and the index of cardio‐electrophysiological balance (iCEB) were calculated in 101 hospitalized COVID‐19 patients and it was compared with 101 non‐COVID‐19 matched controls. A systematic review was performed in four databases and meta‐analysis was conducted using Stata software.

**Results:**

Tp‐Te, TpTe/QT, QRS width, and iCEB were significantly increased in COVID‐19 patients compared with controls (TpTe = 82.89 vs. 75.33 ms (ms), *p*‐value = .005; TpTe/QT = 0.217 vs. 0.203 ms, *p*‐value = .026). After a meta‐analysis of 679 COVID‐19 cases and 526 controls from 9 studies, TpTe interval, TpTe/QT, and TpTe/QTc ratios were significantly increased in COVID‐19 patients. Meta‐regression analysis moderated by age, gender, diabetes mellitus, hypertension, and smoking reduced the heterogeneity. QTd showed no significant correlation with COVID‐19.

**Conclusion:**

COVID‐19 adversely influences the ECG markers of transmural heterogeneity of repolarization. Studies evaluating the predictive value of these ECG markers are warranted to determine their clinical utility.

## 1 INTRODUCTION

Coronavirus disease 2019 (COVID‐19) is caused by the seventh member of the coronavirus family that can infect humans.^1^ The first case of COVID‐19 was detected in Wuhan, China in December 2019 and the disease soon spread across the countries, causing a devastating pandemic. COVID‐19 has been associated with significant cardiovascular comorbidities.^2^ Numerous investigations have been conducted to find the potential risk predictors of major adverse cardiac events.

Direct myocardial damage by the virus, systemic inflammatory state, catecholaminergic response to respiratory distress, adverse effects of therapeutic agents, and deterioration of the underlying cardiovascular disorders can potentially increase the risk of malignant ventricular arrhythmias (VA).^3^ In addition, QT interval prolongation has been seen in patients with COVID‐19 regardless of taking medications that prolong the QT interval.^4^, ^5^, ^6^, ^7^

The role of cardiac muscle repolarization heterogeneity in developing VA has been well studied.^8^ Electrocardiogram (ECG) repolarization markers such as QT dispersion (QTd) and the interval between T wave peak to the T wave end (TpTe) have been suggested as indicators of regional and transmural heterogeneity of myocardial repolarization. These ECG markers have been reported to be greatly influenced by systemic viral infections^9^, ^10^, ^11^ and systemic inflammatory disorders.^12^, ^13^ However, the effect of COVID‐19 on cardiac repolarization is not well established. This study was conducted to investigate the effect of COVID‐19 on various ECG indicators of repolarization heterogeneity.

## 2 METHODS

### 2.1 Patient selection

All consecutive patients who were admitted to Imam Khomeini Hospital Complex, Tehran, Iran with a confirmed diagnosis of COVID‐19 between February 2020 and April 2020 were included in the study. COVID‐19 diagnosis was made either by a positive polymerase chain reaction (PCR) of the nasopharyngeal swab or evident COVID‐19 pneumonia on chest CT scan with no other etiology explaining the imaging findings. The control group was selected from gender‐ and age‐matched patients admitted for elective surgery during the same period of time. The control group had no clinical evidence of COVID‐19 and had negative PCR tests. The patients' demographics, lab results, and admission ECGs were available for all cases and controls. Patients with uninterpretable ECG, complete bundle branch block, nonsinus rhythms (e.g., atrial fibrillation), symptoms of acute coronary syndromes, symptomatic heart failure with ejection fraction (EF) ≤ 40%, outpatient use of QT‐prolonging agents (e.g., fluoroquinolones, azithromycin, etc.) and electrolyte imbalances on admission were excluded. The demographic data and medical history were extracted from the patients' charts. The medications prescribed in the outpatient setting were also collected. The laboratory data including white blood cell count, neutrophil to lymphocyte ratio (NLR), hemoglobin concentration, platelet count, plasma level of C‐reactive protein (CRP), and quantitative levels of troponin T and creatinine were recorded for COVID‐19 cases. This study posed no additive charge or harm to the study population. It was approved by the ethics committee of Tehran University of medical sciences. All patients' identifiers were removed to comply with Health Insurance Portability and Accountability Act regulations. Informed consent was obtained from all patients before the study.

### 2.2 ECG measurements

Surface ECGs were scanned to a personal computer and transferred to Adobe Photoshop CC 2019 software (Adobe Photoshop Version: 20.0.0; Adobe Inc.). The measurement scales were set and the intervals were calculated at 200% magnification.

The QT interval was considered as the distance between the first deflection of the QRS complex and the end of the T wave defined as the intersection of the tangent to the steepest downslope of the T wave and the isoelectric line. The longest QT and QRS intervals were used (usually mid‐precordial V3) to prevent underestimation of isoelectric T waves. In case of a discrete U wave in a precordial lead, that value was omitted.

The TpTe interval was defined as the interval between the T wave peak to the T wave end in lead V5.^14^ All 12‐lead values were recorded and the difference between the longest and the shortest QT and TpTe values in a single beat was defined as QT dispersion and TpTe dispersion, respectively. QT intervals were corrected for heart rate using both Bazett's and Framingham's methods. Bazett's formula was the most common method used by previous studies and this method is the most homogenous method to perform the meta‐analysis and the Framingham's formula is known to be the most accurate one.^15^

To assess the contribution of repolarization heterogeneity duration to the total duration of the action potential, TpTe was divided by QT and QTc and the index of cardio‐electrophysiological balance (iCEB) was defined as the QT to QRS ratio.

### 2.3 Statistical analysis for original study

Continuous variables are presented as mean ± *SD* and categorical variables are reported as frequency and percentage. Student *t* test and *χ* ^2^ test were used to compare continuous variables and categorical variables, respectively. Pearson correlation analysis was applied to assess the correlation of ECG markers with laboratory and clinical data. A 2‐sided *p* value of < .05 was considered statistically significant. Statistical analyses were performed using the SPSS software version 25 (IBM Corp.).

### 2.4 Systematic review search strategy

The current review followed the Preferred Reporting Items for Systematic reviews and Meta‐Analyses (PRISMA) guideline. The review protocol has been published on The International Prospective Register of Systematic Reviews, known as PROSPERO (ID: CRD42021244450). A systematic search was conducted until May 28, 2021 by two independent investigators (E. M. and M. K.) in four databases, including Scopus, Web of Science, PubMed, and Cochrane Library. To address the search question, the search queries included a combination of three keywords without any language restriction or time limitations: ([QT dispersion and its equivalents] OR [TpTe interval and its equivalents]) AND [COVID‐19 and its equivalents]. The complete search queries for each database are described in the Supporting Information Material. A subsidiary manual search was also performed in ResearchGate and Google Scholar.

The studies that presented the mean and standard deviation for any of the four ECG variables including “QTd,” “TpTe,” “TpTe to QT ratio,” and “TpTe to QTc ratio” in COVID‐19 patients were included. All types of observational studies were included. The search results were screened using their titles, abstracts, and full‐texts in more relevant studies. Duplications were removed and the full texts of all the included studies were obtained. The quality of the studies was assessed using Strengthening the Reporting of Observational Studies in Epidemiology (STROBE) checklists at the study level (available as Supporting Information Material). The number of cases and controls, the mean and standard deviation of QTd, TpTe, TpTe/QT, and TpTe/QTc in the study groups, and the characteristics of the study population including age, gender, hypertension, diabetes mellitus, smoking, study location, and COVID‐19 hospitalization status were extracted from each study by two independent investigators. In case of a discrepancy, a consensus was achieved by discussion.

### 2.5 Meta‐analysis of overall data

The values were summarized as mean differences (MDs) with a 95% confidence interval. MDs were standardized using Cohen's method and random‐effects meta‐analysis was performed on each target ECG marker by the Stata software (Stata/MP 16.0; StataCorp LLC). Statistical heterogeneity was evaluated using the Higgins' *I* ^2^ test based on Cochrane's *Q*. Higher *Q* values with *p* values less than .1 were considered as significant heterogeneity. An *I* ^2^ value of <25%, 25%–50%, 50%–75%, and >50% indicated absent, low, moderate, and high heterogeneity, respectively.^16^ Subgroup analysis and meta‐regression were performed in case of severe heterogeneity: 1. Age equal to or above 18 and lower than 18; 2. Male sex more than 55% of the study population, less than 45%, and equal to 50 ± 5%; 3. Country where the study was conducted; 4. Only hospitalized cases were included, only outpatient cases were included, both types of patients were included. Publication bias was evaluated through visual assessment of funnel plots as well as the Egger, Begg, and “trim‐and‐fill” tests with *p* values < .1 indicating publication bias. Sensitivity analysis was performed by excluding individual studies from the meta‐analysis.

## 3 RESULTS

The study population included 101 patients hospitalized due to COVID‐19 and 101 gender‐ and age‐matched controls. Seventy percent of the cases and controls were male with a mean age of 60.11 ± 16.16 years for cases and 61.1 ± 17.22 years for controls. The patients' characteristics are summarized in Table 1.

**Table 1:** Demographic characteristics of our study population

	Case *N* = 101 *N* (% or SD)	Control *N* = 101 *N* (% or *SD*)	*p* value
*Personal history*			
Sex (Male)	71 (70.3)	71 (70.3)	1.000
Age (years)	60.11 (16.16)	61.08 (17.22)	.680
Diabetes Mellitus	41 (40.59)	34 (33.66)	.382
Hypertension	43 (42.57)	36 (35.64)	.387
Stable Ischemic Heart Disease	21 (20.79)	15 (14.85)	.358
Current/Past cigarette smoking	26 (25.74)	39 (38.61)	.301
*Previous medications*			
Aspirin	46 (45.5)	43 (42.6)	.777
Statins	40 (39.6)	40 (39.6)	1.000
ACEI	29 (28.7)	31 (30.7)	.878
Betablocker	38 (37.6)	41 (40.6)	.773
Nitrate	13 (12.9)	8 (7.9)	.357
CCB	7 (6.9)	0	**.014**
Warfarin	3 (3)	2 (2)	1.000
Insulin	9 (8.9)	8 (7.9)	1.000
Diabetes oral agents	34 (33.7)	31 (30.7)	.763
*Laboratory data*			
O2 saturation (%)	91.78 (50–99)		
WBC (10^3^ per microliter)	9.146 (4.971)		
Hb (g/dl)	13.9 (2.3)		
NLR	3.16		
Platelet (10^3^ per microliter)	200.95 (70.00)		
CRP (mg/liter)	18.32 (36.92)		
Baseline Troponin T (ng/ml)	3.802 (2–50,000)		
Creatinine (mg/dl)	1.2 ± 0.7		

*Note*: Bold values are statistically significant (*p* < .05).

Abbreviations: ACEI, angiotensin converting enzyme inhibitor, CCB, calcium channel blocker; CRP, C‐reactive protein; Hb, hemoglobin; NLR, neutrophil to lymphocyte ratio; WBC, white blood cell.

ECG parameters in the study groups are presented in Table 2. The corrected values are based on the Framingham formula. Four markers of repolarization heterogeneity were significantly prolonged in COVID‐19 patients compared with controls, including TpTe (82.89 ± 18.73 vs. 75.33 ± 18.97, *p*‐value = .005), corrected TpTe (96.85 ± 20.90 vs. 88.44 ± 30.09, *p*‐value = .022), TpTe/QT (0.217 ± 0.041 vs. 0.203 ± 0.051, *p*‐value = .026), and QRS width (90.54 ± 13.83 vs. 85.08 ± 14.33 ms, *p*‐value = .007). iCEB and TpTe dispersion were significantly lower in cases (iCEB = 4.27 ± 0.70 vs. 4.54 ± 1.13 ms, *p*‐value = .041; TpTe dispersion = 14.01 ± 11.93 vs. 23.36 ± 16.02 ms, *p*‐value < .001). There were no significant differences in QT dispersion and TpTe/QTc between the two study groups.

**Table 2:** Electrocardiographic characteristics of our study population and the results of mean comparison analysis

Intervals (milliseconds)	Cases mean (*SD*)	Controls mean (*SD*)	*p* value
HR	84 (18)	82 (24)	.557
QTd	36.32 (21.16)	36.40 (21.41)	.979
Corrected QTd	39.25 (25.39)	32.83 (33.90)	.129
TpTe	82.89 (18.73)	75.33 (18.97)	**.005**
TpTec^a^	122.10 (25.95)	108.12 (43.43)	**.006**
TpTe dispersion	14.01 (11.93)	23.36 (16.02)	**<.001**
TpTe/QT	0.217 (0.041)	0.203 (0.051)	**.026**
TpTe/QTc^a^	0.197 (0.0039)	0.187 (0.044)	.102
QRS duration	90.54 (13.83)	85.08 (14.33)	**.007**
iCEB (QT/QRS)	4.27 (0.70)	4.54 (1.13)	**.041**

*Note*: Bold values are statistically significant (*p* < .05).

Abbreviations: HR, heart rate; iCEB, index of cardio‐electrophysiological balance; QTc, corrected QT; QTd, QT dispersion; TpTe, T wave peak to T wave end.

^a^ Corrected by Framingham method.

ECG parameters were also corrected using the Bazett's equation for use in the meta‐analysis. These values are reported separately in Table S1. Possible correlations between ECG markers and laboratory and clinical data were investigated. The oxygen saturation level was positively correlated with the TpTe interval and TpTe/QTc (*r* ^2^ = .28, *p*‐value = .049 for TpTe and *r* ^2^ = .30, *p*‐value = .035 for TpTe/QTc, Figure S1A). The platelet count had a negative correlation with TpTe/QT and TpTe/QTc (*r* ^2^ = −.28, *p*‐value = .021 for TpTe/QT and *r* ^2^ = −.27, *p*‐value = .025 for TpTe/QTc, Figure S1B) and the CRP level had positive correlation with TpTe dispersion (*r* ^2^ = .28, *p*‐value = .025 for TpTe, Figure S1C).

### 3.1 Systematic review and meta‐analysis

**Figure 1:**
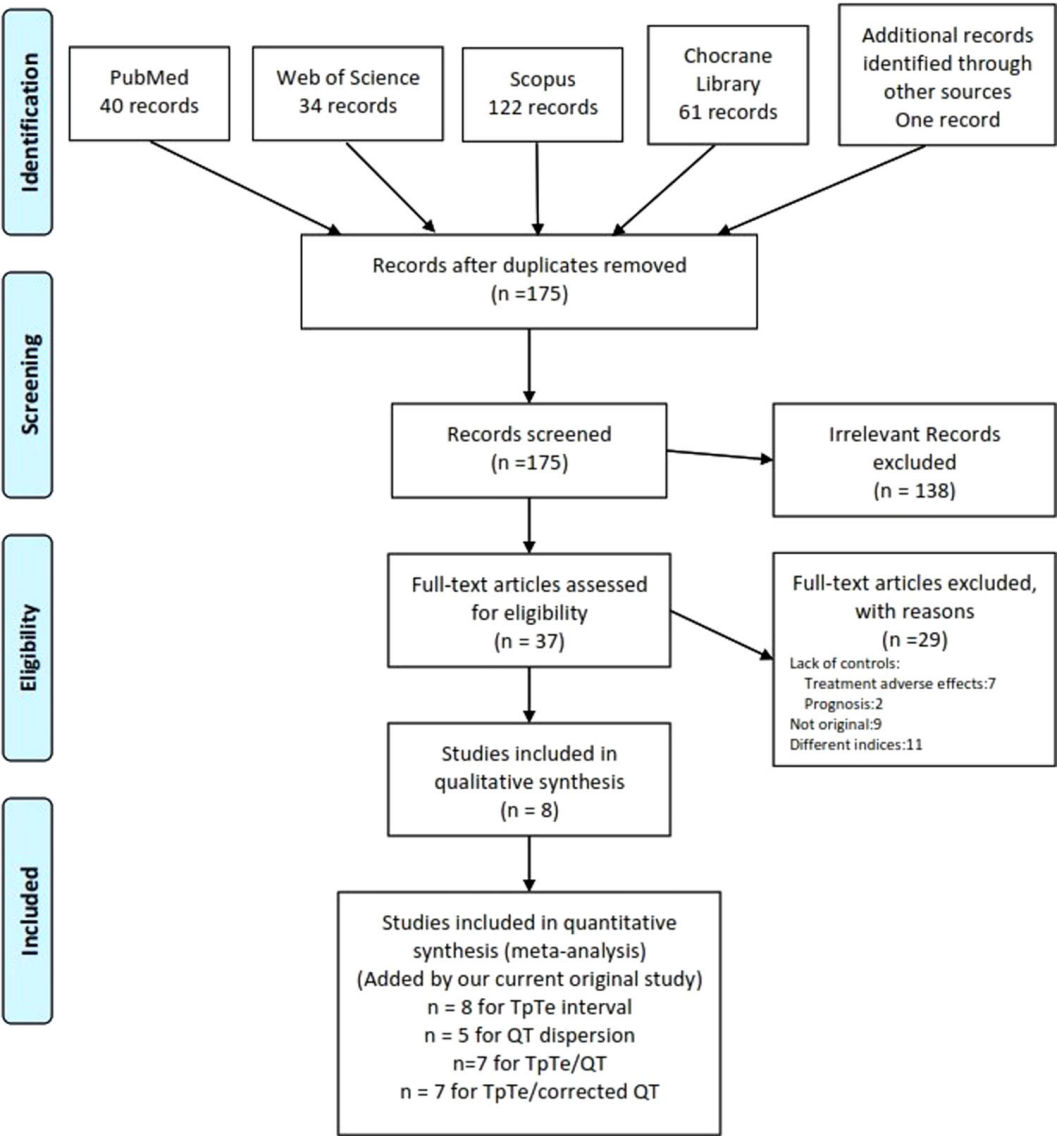
Flowchart showing the screening process of systematic review results

Eight studies were considered eligible for inclusion in the current meta‐analysis and the original data of the present study were added to these eight studies.^4^, ^17^, ^18^, ^19^, ^20^, ^21^, ^22^, ^23^ The flow diagram of the screening process is presented in Figure 1.

After excluding a study by Rubin et al.^4^ (whose characteristics were not reported), the overall mean age of the subjects was 42.43 ± 11.75 years for patients and 42.97 ± 11.70 years for controls. About 58% of the patients and 54% of the controls were male. The prevalence of hypertension, diabetes mellitus, and cigarette smoking was 36%, 27%, and 28% in patients and 29%, 14%, and 21% in controls, respectively. Six studies were performed in six different Turkish cities, one study was conducted in Iraq, one was carried out in the United States, and our study was performed in Iran. The characteristics of the studies are summarized in Table S2.

**Figure 2:**
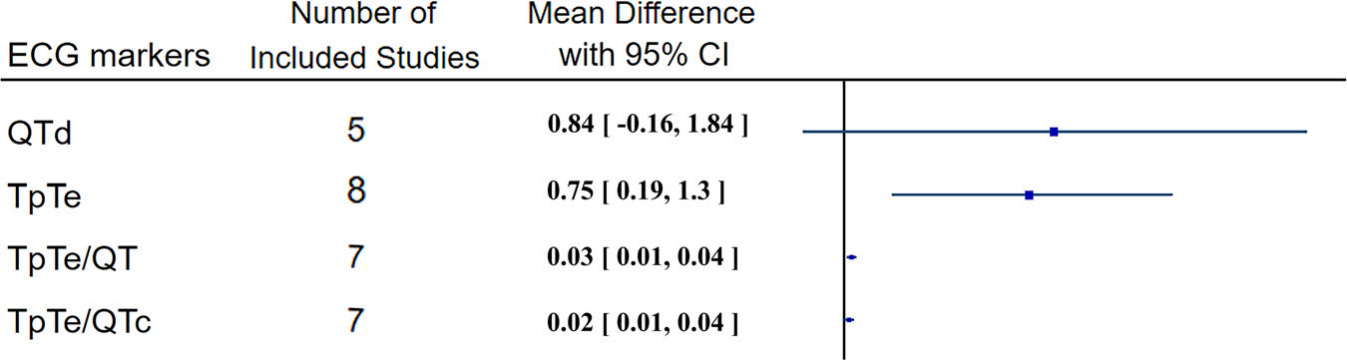
Meta‐analysis of ECG markers of repolarization heterogeneity mean difference between two study groups. CI, confidence interval; COVID‐19, coronavirus disease 2019; MD, mean difference; SD, standard deviation

The overall results of the meta‐analysis are summarized in Figure 2. We observed that the QTd tends to be higher in COVID‐19 cases compared with controls with a *p* value of .1 (QTd SMD = 0.84, *p*‐value = .10, Figure S2); however, there was substantial heterogeneity among studies and subgroup and meta‐regression analysis did not improve it.

The TpTe, TpTe/QT and TpTe/QTc were significantly increased in COVID‐19 patients compared with controls (TpTe SMD = 0.75, *p*‐value = .01, Figure S3; TpTe/QT MD = 0.03, *p*‐value < .01, Figure S4; TpTe/QTc MD = 0.02, *p*‐value = .01, Figure S5). Subgroup analysis based on age, gender predominance, and patient's hospitalization status and meta‐regression moderated by age, sex, diabetes mellitus, hypertension, smoking and hospitalization status reduced the heterogeneity (Figures S6‐S10; Tables S3‐S5).

In the sensitivity analysis of all variables, leaving out individual studies did not change the overall results, the funnel plot was apparently symmetric For TpTe and TpTe/QT and the Egger, Begg, and “trim‐and‐fill” tests revealed no significant publication bias. For QTd, the funnel plot appeared minimally asymmetric, but the Egger and Begg tests revealed a significant publication bias. For TpTe/QTc, the funnel plot was apparently asymmetric and the Egger, Begg, and “trim‐and‐fill” tests revealed a significant publication bias. The trim‐and‐fill test imputed two studies to correct the bias.

## 4 DISCUSSION

According to our original data and the meta‐analysis of previously published data, a significant increase was observed in TpTe and TpTe/QT in COVID‐19 patients. Besides this, there was no significant difference in the QTd between the study groups. To evaluate these electrophysiologic changes in the setting of COVID‐19, we should consider the pathophysiology beyond repolarization heterogeneity, its ECG representators, and their influencing mediators. We previously discussed this concept and its prognostic implications in critical cases of COVID‐19 in our recent letter.^24^ However, despite the strong theoretical logic behind these ECG indicators, there is still a considerable lack of evidence on their application in clinical practice.

VAs are known to be the result of nonhomogenous prolongation of repolarization in different regions of the myocardium rather than a global increase in repolarization time. Dispersion of the QT interval between 12 surface ECG leads has been considered as an indicator of repolarization heterogeneity between the adjacent myocardial regions. At first, reduction in QTd seemed to explain the safety and efficacy of antiarrhythmic agents despite their QT‐prolonging effects, although further studies questioned this.^25^ In terms of COVID‐19, three of the four published studies have reported a significantly increased QTd in these patients, as well as an increased TpTe interval and TpTe/QT^18^, ^20^, ^22^; Interestingly, Yenercag et al. which have reported the most prominent prolongation of TpTe interval in COVID‐19 patients did not observe any significant difference in QTd.^23^ Along with the latter study and our observations, our meta‐analysis revealed the same QTd between COVID‐19 patients and controls. We could not find any considerable difference in the case selection and design of the included studies to explain these controversial results; despite the fact that two of the studies with a significant difference in QTd were conducted on children's age‐group. However, the small number of the included studies makes it unreliable to perform a subgroup analysis based on age.

As the T wave deflection is a representator of electrical gradients between myocardial regions throughout the repolarization, variable measures of T wave interval duration, amplitude, polarity, and the area under the T wave have been proposed to assess repolarization heterogeneity.^26^ TpTe interval was introduced as the interval between termination of repolarization in the first and the last cells across a transmural section of the myocardium (epicardial and M‐cells, respectively).^27^ TpTe and its proportion to QT interval (TpTe/QT) provide a relative (but not absolute^28^) measure of the time interval during which the myocardium is partially repolarized and a “transmural” repolarization heterogeneity is present. The resultant possibility of focal conduction block can be a basis for the generation of malignant VAs,^8^ as it has been reported in various conditions with increased susceptibility to VA.^29^ Along with the results of our meta‐analysis, most of the studies conducted on COVID‐19 cases have reported a significantly increased TpTe and TpTe/QT in these patients.^18^, ^20^, ^21^ The recent study by Colkesen et al. reported the same values of TpTe, TpTe/QT, and TpTe/QTc in COVID‐19 patients and controls, which may be explained by the fact that their case selection was restricted to the outpatient setting.

The TpTe/QTc was increased in COVID‐19 patients in all the published studies, but the difference was not statistically significant for the studies by Colkesen et al. Ozturket al. and our study population. The overall meta‐analysis also showed a significantly increased ratio in COVID‐19 patients. Though this ratio and the corrected TpTe are commonly used as indicators of repolarization heterogeneity there is no logic behind the heart rate correction for TpTe interval and for the nominator or denominator alone in the TpTe/QT ratio. The significant changes observed in these corrected parameters may be due to the more prominent change in the noncorrected baseline values, as it is evident in the results of the current study.

This study also revealed a significant decrease in TpTe dispersion and iCEB in COVID‐19 patients. The clinical and theoretical implication of single‐beat dispersion of TpTe is not yet elucidated. This marker has been suggested to be even more accurate than TpTe alone in predicting the risk of VA,^30^ although we could not explain the significantly lower TpTe dispersion despite the same QT dispersion between the study groups. This observation may reflect a more pronounced TpTe prolongation in leads with shorter baseline TpTe in COVID‐19 patients. Taking all the findings together, the preserved QTd and reduced TpTe dispersion as well as prolonged TpTe in COVID‐19 patients are in favor of increased transmural heterogeneity of repolarization despite a preserved regional heterogeneity.

The reduced iCEB in the setting of COVID‐19 seems to be explained by the significant widening of QRS width in these patients. The QRS widening and reduced iCEB is compatible with the previous study by Alareedh et al. and with the conduction deficits reported in COVID‐19 patients particularly in the Asian population.^3^, ^5^ According to the incidental Asian focus of our included studies, iCEB seems not to be a reliable tool in this population.

Eventually, it should be noted that the study by Alareedh et al. reported the same TpTe and decreased iCEB and TpTe/QTc in COVID‐19 patients which were in contrast with all other studies. As the controversial results were more prominent for two studies by Colkesen et al. who was restricted to outpatient cases and Alareedh et al. who included a combined population of outpatient and hospitalized COVID‐19 cases, reporting the proportion of outpatient cases included in such studies would be greatly helpful to explain the controversial findings.

The observed increase in TpTe and TpTe/QT ratio in COVID‐19 patients is of important value as the repolarization duration of M‐cells is highly influenced by endo/exogenous mediators (e.g., drugs, electrolytes, and catecholamines), which makes the TpTe interval a useful marker to assess temporary electrophysiologic changes in the myocardium. COVID‐19 patients can particularly be complicated with numerous conditions that may affect cardiac electrophysiological properties.

Myocardial injury in the setting of ischemia and viral myocarditis have been previously shown to increase repolarization heterogeneity and prolong the TpTe and TpTe/QT intervals.^8^, ^11^, ^31^ In terms of COVID‐19, cardiac injury is a common complication during hospitalization^32^ and it is not only a cause of death due to myocardial dysfunction and malignant VAs but it is also a prognostic indicator of poor outcomes.^2^ Numerous conditions can exacerbate the cardiac injury in these patients: Inflammatory cytokines storm; Increased myocardial demand secondary to respiratory distress, fever, tachycardia, and thromboembolic obstruction of circulation^33^; and Insufficient coronary supply secondary to hypercoagulable state (acute coronary thrombosis),^34^ coronary vasculitis,^34^, ^35^, ^36^ hypotension, hypoxemia, and possible stress‐induced diffused vasospasms.^37^

Sympathetic activation due to respiratory distress, hypoxia, and fever can increase the markers of repolarization heterogeneity.^38^ In addition, the particular influence of inflammation on repolarization heterogeneity has been previously ascertained in patients suffering from systemic inflammatory disorders^12^, ^13^, ^39^, ^40^ and the TpTe interval has been correlated with plasma levels of inflammatory biomarkers in HIV patients.^9^ In the setting of COVID‐19, the CRP level was correlated with TpTe and TpTe/QT ratio in studies conducted by Yenercag et al.^23^ and Koc et al.^21^; however, the results of a study by Colkesen et al.^19^ and our findings did not indicate such a correlation. Our current study also found a significant positive correlation between TpTe dispersion and plasma CRP level and a nonsignificant tendency of the TpTe dispersion to be higher in patients with increased NLR (*r* ^2^ = .185, *p*‐value = .128). In addition, TpTe/QT and TpTe/QTc ratios were negatively correlated with the platelet count, which can be secondary to bone marrow suppression and the resultant thrombocytopenia in the setting of septicemia and systemic inflammation.

**Figure 3:**
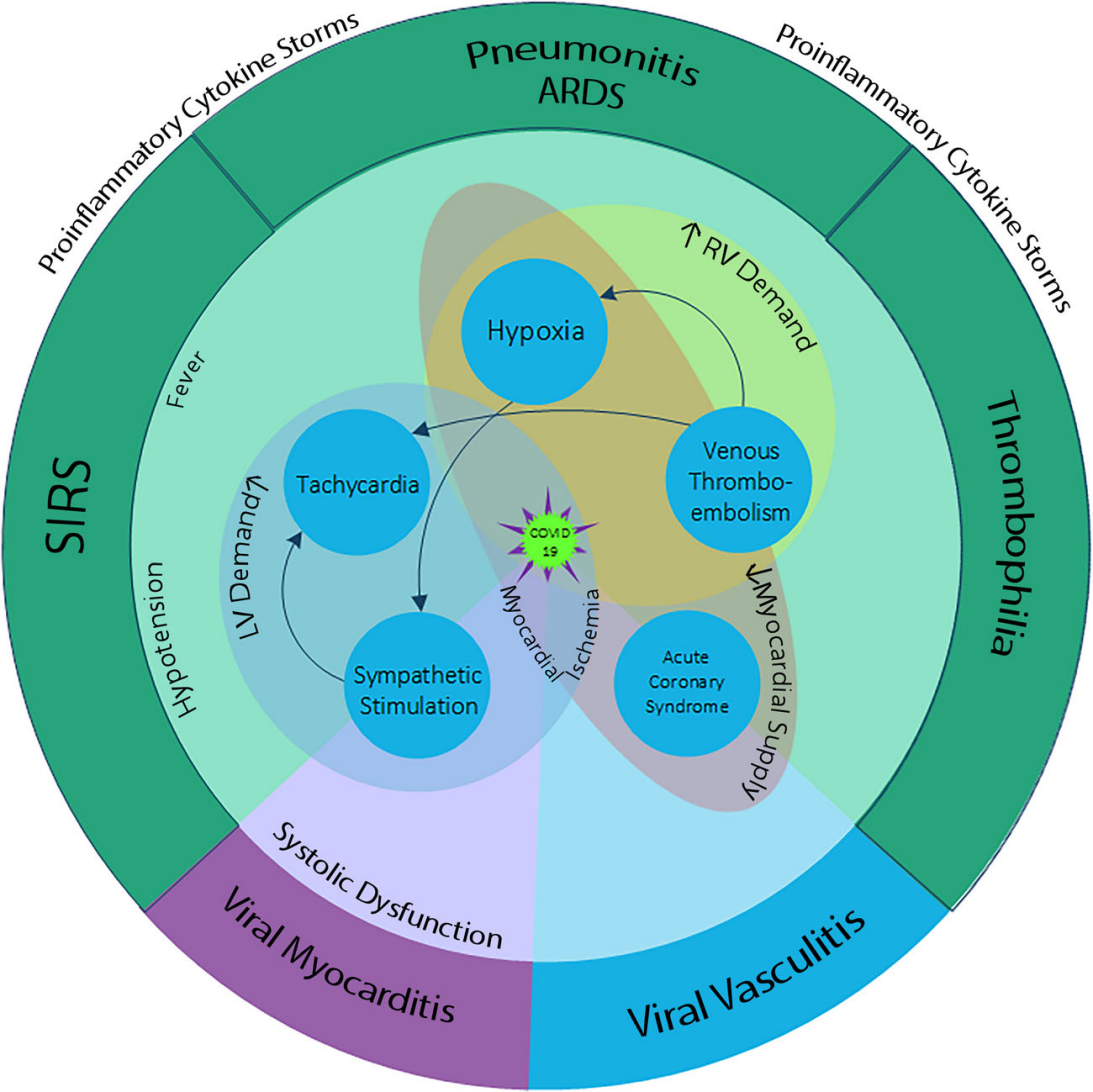
A schematic description of variables affecting on cardiac electrical stability in the course of COVID‐19. (The flash sign represents adrenergic stimulation, ARDS, acute respiratory distress syndrome; SIRS, systemic inflammatory response syndrome, PTE, pulmonary thromboembolism, ACS, acute coronary syndrome, RV, right ventricle, TpTe, T wave peak to T wave end)

The multifaceted effects of COVID‐19 on myocardial repolarization heterogeneity markers are illustrated in Figure 3. Together with the focus on the pathophysiology of COVID‐19, the baseline characteristics of patients should be also cautiously explored to prevent misconceptions due to their confounding effects on the ECG markers.^41^ The age is a positive moderator of the TpTe and TpTe/QT intervals in low‐risk populations.^42^ This association was not found in our study population, which may be caused by the fact that even our controls were not selected from a low‐risk population.

In terms of gender‐specific differences in myocardial electrophysiologic properties, androgen deprivation and anti‐androgen treatment have been associated with a prolonged TpTe interval and administration of testosterone can shorten this interval.^43^ In addition, women have shown greater drug‐induced prolongation in the TpTe interval.^44^ We did not observe a direct correlation between the gender status of subjects and their ECG values; although subgroup analysis of studies with male predominance considerably reduced between‐study heterogeneity.

Among the common comorbidities, diabetes mellitus has been associated with a tendency to increase the TpTe dispersion and TpTe/QT ratio.^45^ LV hypertrophy, diastolic dysfunction, LV mass index, and elevated systemic blood pressure are also associated with an increase in the TpTe interval and TpTe/QT ratio.^46^, ^47^ Moreover, chronic tobacco users develop a transient increase in TpTe interval following each episode of cigarette smoking.^48^ As the studies included in the present meta‐analysis had heterogeneous populations in terms of age, sex, and the mentioned comorbidities (Table S2), meta‐regression analysis moderated by these factors considerably improved the heterogeneity.

Eventually, the drug‐induced changes in repolarization heterogeneity markers and their role in the development of VA are well studied^49^ and so is the repolarization heterogeneity associated with malignant VA in the setting of acute coronary events.^31^ In this study, these confounding factors were relatively corrected by excluding the affected cases. The same exclusion strategy was followed by all the studies included in our meta‐analysis except for two studies by Ece et al.^20^ and Cevik et al.,^18^ which were conducted in pediatric age groups.

## 5 CONCLUSION

According to this study, novel indicators of repolarization heterogeneity including TpTe, TpTe/QT, and TpTe/QTc are considerably increased in the course of COVID‐19. These adverse electrophysiological changes can be secondary to numerous conditions that impair myocardial supply‐demand, stimulate myocardial sympathetic nerves, and directly injure the myocytes in the course of COVID‐19. Owing to the imposed burden of cardiac arrhythmias in COVID‐19 morbidity and mortality, measurement of the electrocardiographic markers of repolarization heterogeneity may be considered an available, cost‐effective, and robust method of risk stratification, particularly in more severe cases.

### 5.1 Limitations

ECG measurements are relatively heterogeneous and operator‐dependent. Besides this, there is no expert consensus on the definition of most of the novel markers we addressed in this study. Same heterogeneities exist about different methods of COVID‐19 diagnosis and the criteria for hospitalization. Another challenge was to prepare a sufficient sample of healthy controls from the low‐risk population with an available ECG, negative COVID‐19 PCR, and matched age and sex, which was not feasible, timely, and cost‐efficacious. So that we chose a sample of elective patients admitted to the surgery ward whose COVID‐19 was rolled out to prevent complications and they all had ECG by routine. In addition, investigating the prognostic value of ECG markers in predicting the in‐hospital or long‐term adverse cardiovascular events including arrhythmias and mortality could have been of great value but a larger sample of patients was needed to perform a statistically reliable analysis of these relatively rare outcomes.

## 6 CLINICAL PERSPECTIVE

- According to previous studies, cardiovascular complications have imposed considerable burden to patients with COVID‐19.
- Repolarization heterogeneity is increased in patients with COVID‐19, before initiation of QT‐prolonging agents.
- Myocarditis, inflammation, Thrombophilia, Coronary ischemia, Hypoxia, adrenergic stimulation and therapeutic agents are possible mediators influencing repolarization duration and heterogeneity in patients with COVID‐19.
- TpTe, TpTe/QT, QRS width and iCEB should be considered as risk markers of arrhythmia in patients with COVID‐19.

## CONFLICT OF INTERESTS

The authors declare that there are no conflict of interests.

## AUTHOR CONTRIBUTIONS

Pejman Mansouri, Shayan Mirshafiee, Behnam Hedayat, and Mojtaba Salarifar provided the original data summarized in Tables 1 and 2. The systematic review, data extraction, and meta‐analysis of extracted data were conducted by Elham Mahmoudi and Mohammad Keykhaei. Matthew F. Yuyun supervised the statistics and Elham Mahmoudi wrote the majority of manuscript. Reza Mollazadeh was the corresponding author who supervised all the process and critically appraised, edited, and finalized the final manuscript in partnership with Hirad Yarmohammadi and Matthew F. Yuyun.

## Supporting information

Supporting information.

## Data Availability

The data that support the findings of this study are available on request from the corresponding author. The data are not publicly available due to privacy or ethical restrictions.
