# Supplementary material for: Ventricular repolarization heterogeneity in patients with COVID‐19: Original data, systematic review, and meta‐analysis
Source: Clin Cardiol. 2022 Jan 10;45(1):110–8. doi: 10.1002/clc.23767 (PMC8799060; doi:10.1002/clc.23767)
Supplement: Supplementary file 1 — Supporting information. [file CLC-45--s001.docx]

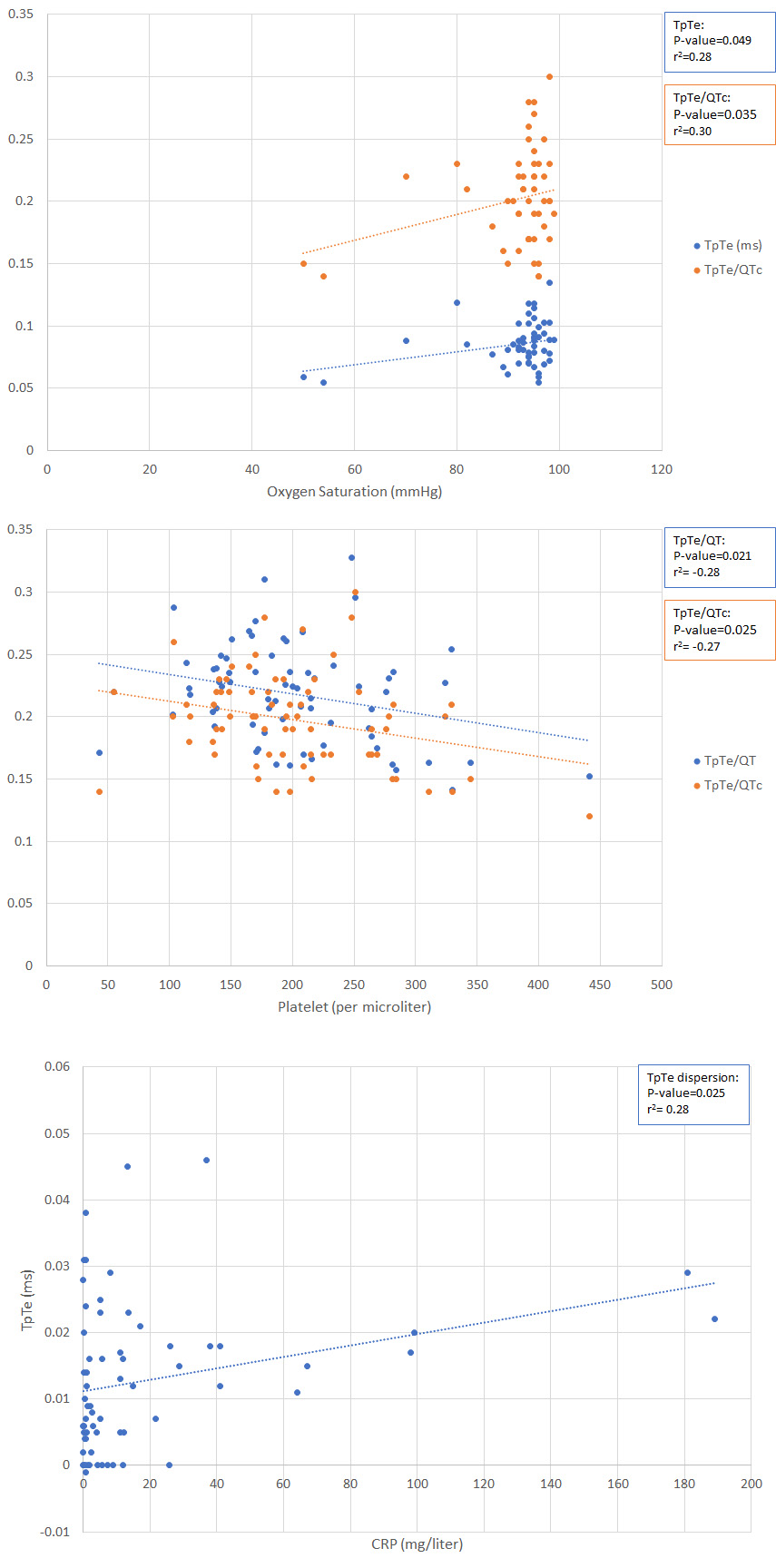


Supplementary Figure 1. Correlation between electrocardiographic markers of repolarization heterogeneity and A) Oxygen saturation, B) platelet and C) C-reactive protein (CRP)


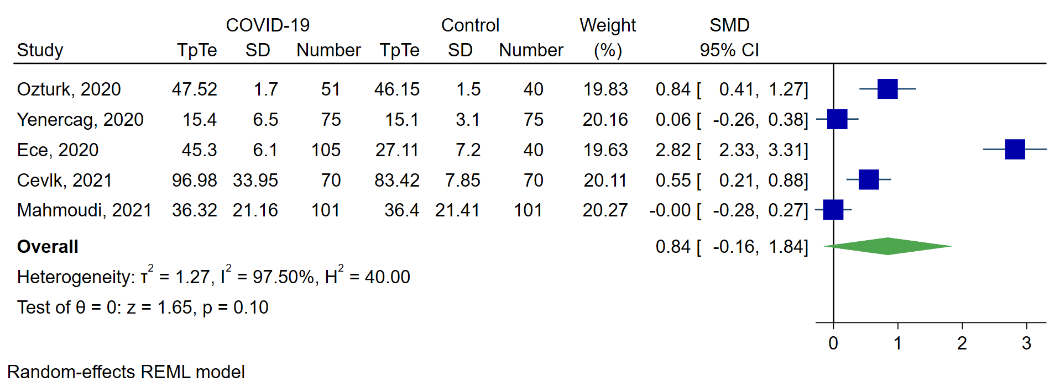


Supplementary Figure 2. Meta-analysis of QT interval dispersion standardized mean difference

COVID-19= Corona virus disease-2019, SD=standard deviation, SMD-standardized mean difference, CI= confidence interval


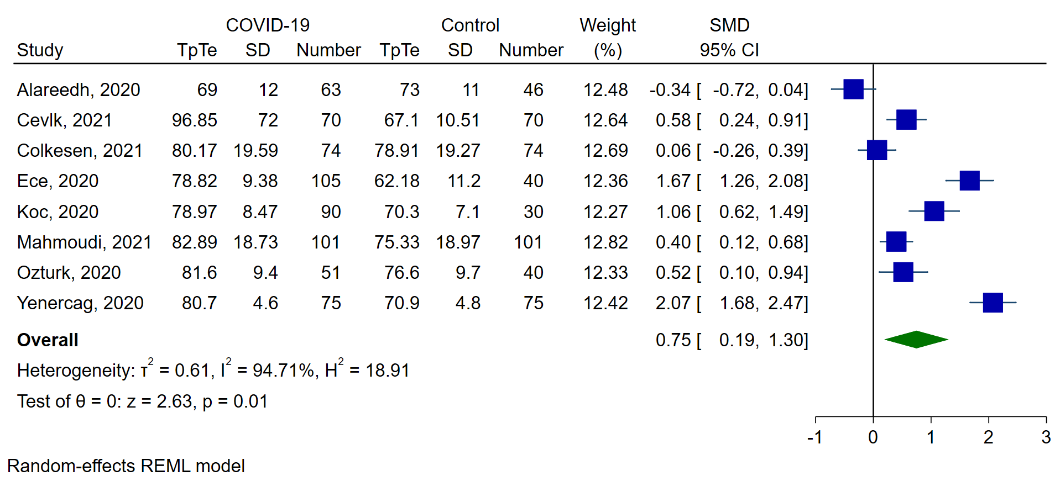


Supplementary Figure 3. Meta-analysis of peak of T wave to end of T wave standardized mean difference

COVID-19= Corona virus disease-2019, SD=standard deviation, SMD=standardized mean difference, CI= confidence interval


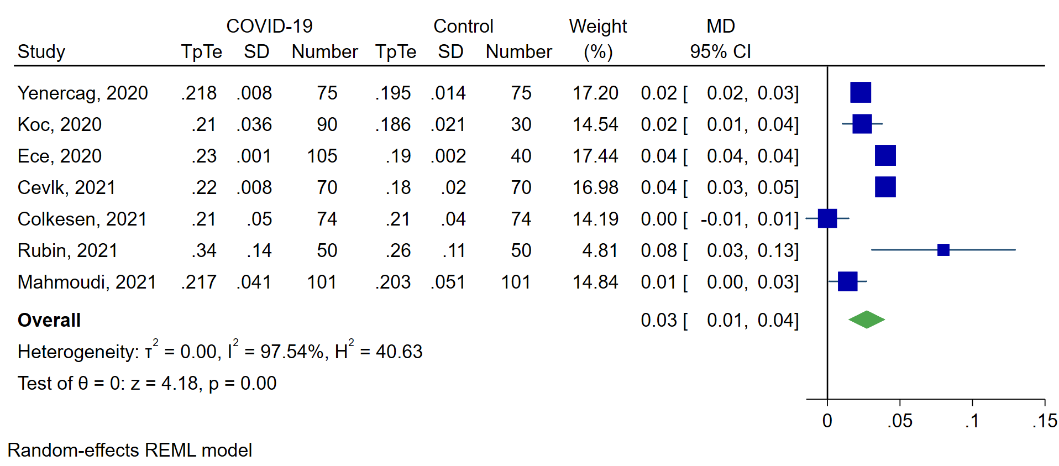


Supplementary Figure 4.Meta-analysis of peak of T wave to end of T wave to QT interval ratio mean difference

COVID-19= Corona virus disease-2019, SD=standard deviation, MD= mean difference, CI= confidence interval


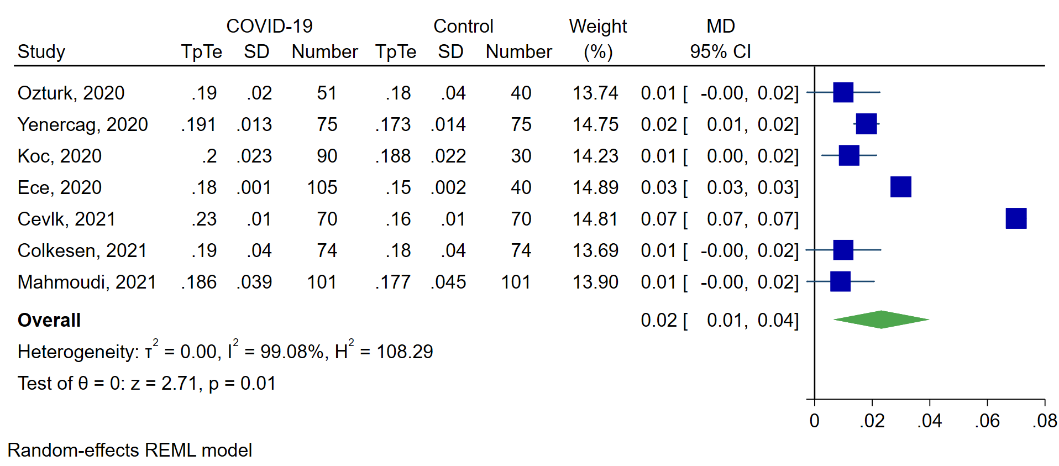


Supplementary Figure 5. Meta-analysis of peak of T wave to end of T wave to corrected QT interval ratio mean difference

COVID-19= Corona virus disease-2019, SD=standard deviation, MD= mean difference, CI= confidence interval

Supplementary Figure 6. Subgroup meta-analysis of peak of T wave to end of T wave standardized mean difference based on patients hospitalizetion.

COVID-19= Corona virus disease-2019, SD=standard deviation, SMD=standardized mean difference, CI= confidence interval

Supplementary Figure 7. Subgroup meta-analysis of peak of T wave to end of T wave standardized mean difference based on male predominance in study population

COVID-19= Corona virus disease-2019, SD=standard deviation, SMD=standardized mean difference, CI= confidence interval

Supplementary Figure 8. Subgroup meta-analysis of peak of T wave to end of T wave to QT interval ratio mean difference based on male predominance in study population

COVID-19= Corona virus disease-2019, SD=standard deviation, MD= mean difference, CI= confidence interval

Supplementary Figure 9. Subgroup meta-analysis of peak of T wave to end of T wave to corrected QT interval ratio mean difference based on age

COVID-19= Corona virus disease-2019, SD=standard deviation, MD= mean difference, CI= confidence interval

Supplementary Figure 10. Subgroup meta-analysis of peak of T wave to end of T wave to corrected QT interval ratio mean difference based on male predominance in study population

COVID-19= Corona virus disease-2019, SD=standard deviation, MD=mean difference, CI= confidence interval

Supplementary Table 1. Electrocardiographic characteristics of our study population and the results of mean comparison analysis

| Intervals in milliseconds | Cases  Mean (SD) | Controls  Mean (SD) | P-value |
| --- | --- | --- | --- |
| HR | 84 (18) | 82 (24) | 0.557 |
| QTd | 36.32 (21.16) | 36.40 (21.41) | 0.979 |
| Corrected QTd | 42.41 (24.73) | 42.07 (24.17) | 0.922 |
| TpTe | 82.89 (18.73) | 75.33 (18.97) | **0.005** |
| TpTec* | 96.85 (20.90) | 88.44 (30.09) | **0.022** |
| TpTe dispersion | 14.01 (11.93) | 23.36 (16.02) | **<0.001** |
| TpTe/QT | 0.217 (0.041) | 0.203 (0.051) | **0.026** |
| TpTe/QTc* | 0.186 (0.039) | 0.177 (0.045) | 0.118 |
| QRS duration | 90.54 (13.83) | 85.08 (14.33) | **0.007** |
| iCEB (QT/QRS) | 4.27 (0.70) | 4.54 (1.13) | **0****.041** |

*Corrected by Bazett’s method

HR=Heart Rate, QTd=QT dispersion, QTc= corrected QT, TpTe=T wave peak to T wave end,iCEB=index of cardio-electrophysiological balance

Supplementary Table 2. Baseline characteristics of studies included in meta-analysis

| Study | Study location | Hospitalization status | COVID-19 cases | Non-COVID controls | Age  Years (SD) | Male Sex (%) | Hypertension (%) | Diabetes Mellitus (%) | Smoking (%) |
| --- | --- | --- | --- | --- | --- | --- | --- | --- | --- |
| Alareedh et al. | Iraq | Combined hospitalized and outpatient COVID-19 patients | 63 | 46 | 54.00 (12) | 43 | 37 | 17 | 12 |
| Cevik et al. | Turkey | Only hospitalized patients | 70 | 70 | 8.31 (6.7) | 51 | N/R | N/R | N/R |
| Colkesen et al. | Turkey | Only outpatient clinic visits | 74 | 74 | 51.67 (11.93) | 48 | 24 | 13 | 10 |
| Ece et al. | Turkey | Combined hospitalized and outpatient COVID-19 patients | 105 | 40 | 11.00 (1.2) | 43 | N/R | N/R | N/R |
| Koc et al. | Turkey | Combined hospitalized and outpatient COVID-19 patients | 90 | 30 | 63.62 (12.85) | 71 | 21 | 13 | 22 |
| Mahmoudi et al. | Iran | Only hospitalized patients | 101 | 101 | 60.59 (16.69) | 70 | 39 | 28 | 32 |
| Ozturk et al.[19] | Turkey | Only hospitalized patients | 51 | 40 | 48.55 (15.80) | 61 | 11 | 9 | 16 |
| Yenercag et al.[20] | Turkey | Combined hospitalized and outpatient COVID-19 patients | 75 | 75 | 52.85 (16.85) | 53 | 53 | 34 | 38 |
| Overall | N/A | N/A | 629 | 476 | 40.28 (10.83) | 53 | N/A | N/A | N/A |

The study by Rubin et al. is not included (refer to the text).

N/R= not reported, N/A=not applicable

Supplementary Table 3. Meta-regression analysis to define sources of heterogeneity (for TpTe interval)

| Variable | Coefficient | SE | Z | P-value | 95% CI | | |
| --- | --- | --- | --- | --- | --- | --- | --- |
| Age | -0.0229204 | 0138257 | -1.66 | 0.097 | -0.0500181 | 0.0041774 |  |
| Sex | -0.027092 | 0.0341638 | -0.79 | 0.428 | -0.0940518 | 0.0398678 |  |
| Diabetes Mellitus | 0.0705128 | 0.1946684 | 0.36 | 0.717 | -0.3110304 | 0.4520559 |  |
| Hypertension | -0.0902293 | 0.078657 | -1.15 | 0.251 | -0.2443942 | 0.0639355 |  |
| Smoking | 0.1000146 | 0.0960001 | 1.04 | 0.297 | -0.0881422 | 0.2881713 |  |
| Hospitalization | 0.5322952 | 0.2613803 | 2.04 | 0.042 | 0.0199992 | 1.044591 |  |
| Constant | 1.572495 | 1.954544 | 0.80 | 0.421 | -2.258342 | 5.403331 |  |

Residual I^2^=64.01%, Q=2.78, P-value=0.095, TpTe=T wave peak to T wave end

SE=standard error, CI=confidence interval

Supplementary Table 4. Meta-regression analysis to define sources of heterogeneity (for TpTe/QT)

| Variable | Coefficient | SE | Z | P-value | 95% CI | |
| --- | --- | --- | --- | --- | --- | --- |
| Age | -0.0011353 | 0.000247 | -4.60 | 0.000 | -0.0016194 | -0.0006513 |
| Sex | 0.0012303 | 0.0004282 | 2.87 | 0.004 | 0.000391 | 0.0020696 |
| Diabetes Mellitus | 0.0005274 | 0.0002302 | 2.29 | 0.022 | 0.0000763 | 0.0009785 |
| Hospitalization | 0.0069282 | 0.0022825 | 3.04 | 0.002 | 0.0024546 | 0.0114018 |
| Constant | -0.0213754 | 0.0225081 | -0.95 | 0.342 | -0.0654906 | 0.0227397 |

Residual I2=0.09%, Q=0.50, P-value=0.479, TpTe=T wave peak to T wave end

SE=standard error, CI=confidence interval

Supplementary Table 5. Meta-regression analysis to define sources of heterogeneity (for TpTe/QTc)

| Variable | Coefficient | SE | Z | P-value | 95% CI | |
| --- | --- | --- | --- | --- | --- | --- |
| Age | -0.0017267 | 0.0006045 | -2.86 | 0.004 | -0.0029114 | -0.0005419 |
| Sex | 0.0016153 | 0.0010157 | 1.59 | 0.112 | -0.0003754 | 0.003606 |
| Diabetes Mellitus | - 0.0059244 | 0.0042615 | -1.39 | 0.164 | -0.0142767 | 0.0024279 |
| Hypertension | 0.0044973 | 0.0029792 | 1.51 | 0.131 | -0.0013417 | 0.0103363 |
| Hospitalization | -0.0063112 | 0.0067596 | -0.93 | 0.350 | -0.0195598 | 0.0069374 |
| Constant | 0.0036526 | 0.0467123 | 0.08 | 0.938 | -0.0879017 | 0.095207 |

Residual I^2^=88.83, Q=8.96, P-value=0.0028, TpTe=T wave peak to T wave end, QTc= corrected QT

SE=standard error, CI=confidence interval
